# The Louisville Medical Institute

**Published:** 1841-08

**Authors:** H. Miller

**Affiliations:** Dean


### THE LOUISVILLE MEDICAL INSTITUTE.

As usual, the dissecting rooms will be opened on the first of October; and in addition to giving the ordinary instructions in dissecting, the Demonstrator of Anatomy will deliver, during the month, a course of lectures on Surgical Anatomy. The Professor of Surgery also, will deliver lectures on diseases of the eye, strabismus, and club-foot. We think that students will find it greatly to their advantage to spend a month in this way previous to the commencement of the regular lectures. H. Miller, *Dean.*

